# Glutathione Induced Immune-Stimulatory Activity by Promoting M1-Like Macrophages Polarization via Potential ROS Scavenging Capacity

**DOI:** 10.3390/antiox8090413

**Published:** 2019-09-18

**Authors:** Da Hye Kwon, Hyesook Lee, Cheol Park, Su-Hyun Hong, Sang Hoon Hong, Gi-Young Kim, Hee-Jae Cha, Suhkmann Kim, Heui-Soo Kim, Hye-Jin Hwang, Yung Hyun Choi

**Affiliations:** 1 Anti-Aging Research Center and Department of Biochemistry, College of Korean Medicine, Dong-eui University, Busan 47340, Korea; 14711@deu.ac.kr (D.H.K.); 14769@deu.ac.kr (H.L.); hongsh@deu.ac.kr (S.-H.H.); 2 Department of Molecular Biology, College of Natural Sciences, Dong-eui University, Busan 47340, Korea; parkch@deu.ac.kr; 3 Department of Korean Internal Medicine, College of Korean Medicine, Dong-eui University, Busan 47227, Korea; shhong@deu.ac.kr; 4 Department of Marine Life Sciences, School of Marine Biomedical Sciences, Jeju National University, Jeju 63243, Korea; immunkim@jejunu.ac.kr; 5 Department of Parasitology and Genetics, College of Medicine, Kosin University, Busan 49104, Korea; hcha@kosin.ac.kr; 6 Department of Chemistry, College of Natural Sciences, Pusan National University, Busan 46241, Korea; suhkmann@pusan.ac.kr; 7 Department of Biological Sciences, College of Natural Sciences, Pusan National University, Busan 46241, Korea; khs307@pusan.ac.kr; 8 Department of Food and Nutrition, College of Nursing, Healthcare Sciences & Human Ecology, Dong-eui University, Busan 47340, Korea; hhj2001@deu.ac.kr

**Keywords:** glutathione, reactive oxygen species, heme oxygenase-1, immune response, macrophage polarization, inflammatory cytokines, antioxidant

## Abstract

The present study investigated the immunomodulatory activity of reduced glutathione (GSH) by assessment of the macrophage polarization (MP)-mediated immune response in RAW 264.7 cells. Furthermore, we identified the signal pathway associated with immune regulation by GSH. The expressions of MP-associated cytokines and chemokines were assessed using cytokine array, nCounter Sprit platform, ELISA and immunoblotting. Phagocytosis activity and intracellular reactive oxygen species (ROS) generation were measured using fluorescence-activated cell sorter. As results of the cytokine array and nCounter gene array, GSH not only up-regulated pro-inflammatory cytokines, including interleukins and tumor necrosis factor-α, but also overexpressed neutrophil-attracting chemokines. Furthermore, GSH significantly stimulated the production of immune mediators, including nitric oxide and PGE_2_, as well as phagocytosis activity through nuclear factor kappa B activation. In addition, GSH significantly decreased LPS-induced ROS generation, which was associated with an activation of nuclear factor erythroid-derived 2-related factor 2 (Nrf2)/ heme oxygenease-1 (HO-1) signaling pathway. Our results suggest that GSH has potential ROS scavenging capacity via the induction of Nrf2-mediated HO-1, and immune-enhancing activity by regulation of M1-like macrophage polarization, indicating that GSH may be a useful strategy to increase the human defense system.

## 1. Introduction

The immune system is a series of effector mechanisms that can destroy pathogens, such as fungi, viruses, bacteria, and parasites [1]. Macrophages are large phagocytic cells, and play an important regulator in the innate and adaptive immune response to pathogens [2]. In response to environmental signals, macrophages induce macrophage polarization (MP), a process of different functionally distinct phenotypes, such as classically activated macrophages (M1), and alternatively activated macrophages (M2) [3]. M1 polarized macrophages play a host defense against pathogens, and they are considered to promote the type 1 T helper (Th1) immune response [4]. They have strong microbicidal, pro-inflammatory, and tumoricidal activity, and are characterized by the up-regulation of pro-inflammatory cytokines (tumor necrosis factor (TNF)-α, interleukin (IL)-1β IL-6, IL-12 and IL-23), chemokines (C-X-C motif chemokine ligand (CXCL) 1-3, CXCL-5 and CXCL8-10), and reactive nitrogen species or reactive oxygen species (ROS) [5,6,7,8]. In contrast, M2 macrophages induce tissue remodeling, angiogenesis, and tumor progression, and are considered to promote the Th2 immune response [9]. M2a and M2c phenotypes, induced by either the Th2 cytokines IL-4 or IL-10, respectively, have anti-inflammatory effect via the up-regulation of anti-inflammatory cytokines [10]. The distinguishing features of M2 polarized macrophages were high levels of expression of cluster of differentiation (CD) 16, (C-X-C motif) chemokine receptor (CXCR) 1, CXCR2, and (C-C motif) chemokine receptor (CCR) 2, and efficient phagocytic activity [5,6,7,8]. The imbalance of MP is resulted from their conflicting roles for inflammation and immune system, and that have effect on influencing various diseases, such as obesity, cancer, and rheumatoid arthritis [8]. Therefore, the stimulation of the immune system via regulation to M1 polarized macrophages is one of the important strategies to increase the human defense system.

Glutathione is composed of three amino acids of l-cysteine, l-glutamic acid, and glycine, and acts as an antioxidant [11]. Glutathione modulates the leukotriene regulation, prostaglandin metabolism, cell proliferation, and regulation of the immune responses [12]. Especially, the balance of intracellular reduced glutathione (GSH) and oxidized glutathione plays an essential role in controlling the cellular immune response. [13,14]. As is well known, low levels of GSH decrease the level of secretion of IL-12, and lead to polarization from the Th1 cytokine profile toward Th2 response patterns, but high levels of GSH favor a Th1 response [15,16]. Furthermore, numerous studies identified that GSH deficiency occurs, and is a critical aspect that can contribute to the imbalance in the Th1/Th2 response [17,18,19,20,21]. One study reported that the recovery of GSH levels can undermined the pathology by down-regulation of the Th1 immune response [22]. Although GSH improved Th1 immune response, it remains poorly understood whether it has effects on the regulation of MP in RAW 264.7 macrophages. Therefore, in the present study, we investigated the immunomodulatory activity of GSH by assessment of the M1-mediated immune response, including MP-related genes expression, nitric oxide (NO) and prostaglandin E2 (PGE2) production, phagocytosis activity, and ROS generation in RAW 264.7 macrophages. Moreover, we studied the signal pathway associated with the immunomodulatory activity of GSH.

## 2. Materials and Methods

### 2.1. Chemicals and Reagents

Reduced glutathione (Luthione^®^) was obtained from Daehan New Pharm. Co., Ltd. (Seoul, Republic of Korea). Dulbecco’s modified Eagle’s medium (DMEM), RPMI 1640 GlutaMAX^TM^ medium, fetal bovine serum (FBS), and Dulbecco’s phosphate buffered saline (DPBS) were obtained from WelGENE Inc. (Daegu, Republic of Korea). Lipopolysaccharides (LPS; Escherichia coli Serotype, 055:B5), sulfanilamide, N-(1-Naphthyl) ethylenediamine dihydrochloride (NED), and phosphoric acid were purchased from the Sigma-Aldrich Chemical Co. (St. Louis, MO, USA). 3-(4,5-Dimethylthiazol-2-yl)-2,5-diphenyltetra-zolium bromide (MTT) and 5,6-carboxy-2′,7′-dichlorodihydrofluorescein diacetate (DCF-DA) were obtained from Invitrogen (Carlsbad, CA, USA). Mouse cytokine array kit (item No. ARY006), interleukin (IL)-1β (item. No. MLB00C), IL-4 (item No. M4000B), IL-10 (item No. M1000B) and tumor necrosis factor (TNF)-α (item No. MTA00B) enzyme-linked immunosorbent assay (ELISA) kit were purchased from R&D Systems, Inc. (Minneapolis, MN, USA). PGE2 (item No. 500141) enzyme-linked immunosorbent assay (ELISA) kit and phagocytosis assay kit (item No. 500290) were obtained from Cayman Chemical (Ann Arbor, MI, USA). The Bradford Protein assay kit was purchased from Bio-Rad Laboratories (Hercules, CA, USA). Table 1 describes the primary and secondary antibodies used for immunoblotting. All other chemicals used were of analytical grade, and were purchased from the Sigma-Aldrich Chemical Co.

### 2.2. Cell Culture

Murine macrophage-like RAW 264.7 cells were obtained from the Korea Cell Line Bank (Seoul, Republic of Korea), and were cultured in DMEM supplemented with 10% FBS. Human monocytic cell line THP-1 and U-937 cells were purchased from American Type Culture Collection (Manassas, MD, USA) and maintained in RPMI 1640 GlutaMAX^TM^ medium supplemented with 10% FBS. The culture medium was replaced every 2–3 days, and maintained in an incubator at 37 °C in an atmosphere of 5% CO_2_. For all experiments, the RAW264.7 cells were grown to 80–90% confluence.

### 2.3. Assessment of Cell Viability

The cell viability was determined by MTT assay. Briefly, RAW 264.7 cells were seeded on 96-well plates at a density of 1 × 10^4^ cells/well, and incubated for 24 h. The cells were treated with different concentrations of GSH of 0.5–2.0 mg/mL and LPS of 1–2 ng/mL. After 24 h, MTT solution was added to each well, and incubated for 2 h at 37 °C. The medium was discarded, and then dimethylsulfoxide was added to dissolve the formazan dye. The absorbance was measured using a microplate reader (VERSA Max; Molecular Device Co., Sunnyvale, CA, USA) at 540 nm. The morphological changes of cells were visualized with phase-contrast microscopy (Carl Zeiss, Oberkochen, Germany).

### 2.4. Cytokines Profiling

The relative expression levels of 40 mouse cytokines were determined in cell culture supernatant using a mouse cytokine array kit. In brief, RAW 264.7 cells were incubated with control media, 0.5, or 1.0 mg/mL GSH for 24 h, and LPS (1 ng/mL) was used as a positive control [23]. Following this, the supernatant was incubated on a nitrocellulose membrane containing 40 different cytokine antibodies for 1 h. The membranes were washed, and then incubated with streptavidin-horseradish peroxidase (HRP) for 30 min. Chemiluminescent detection was performed according to the manufacturer’s instructions. Immune-reactive spots were visualized by the Fusion FX Image system (Vilber Lourmat, Torcy, France), and quantitative analysis of spots was performed using the ImageJ^®^ software (version 1.50i; NIH, Bethesda, MD, USA) to quantify protein expression levels.

### 2.5. nCounter Gene Expression Assay

We applied the nCounter in-solution hybridization method using nCounter Sprint platform (NanoString Technologies, Inc. Seattle, WA, USA) to measure the gene expression levels of candidate genes, as previously described [24]. The cells were treated with GSH or LPS, and incubated for 24 h, and then the total RNAs isolated by collecting the cells. After solution-phase hybridization between the target mRNA and reporter-capture probe pairs, excess probes were removed, and the probe/target complexes were aligned and immobilized in the nCounter cartridge (NCT-120), which was then placed in a digital analyzer for image acquisition and data processing. The raw data was normalized using the housekeeping gene, and the gene expression change was represented by heatmap. The heatmap represented differentially expressed genes by RNA sequencing analysis with fold-change cutoff of 0.5 and 2 (red and green, respectively).

### 2.6. Measurement of the NO, IL-1β, IL-4, IL-10, TNF-α, and PGE_2_ Production

The cells were treated with 0.5 or 1.0 mg/mL GSH for 24 h, and then cell culture supernatants were harvested to measure the NO, cytokines, and PGE_2_ production. LPS (1 ng/mL) and DMEM were used as the positive control and blank control, respectively. The production of NO was assayed using Griess reagent, as previously described [25]. In brief, 100 μL of sulfanilamide/NED solution was mixed with an equal volume of supernatant, and incubated for 5 min at room temperature (RT) in dark. The absorbance was measured using a microplate reader at 540 nm. The nitrite concentration of samples was calculated using the standard curve. The amount of IL-1β, IL-4, and IL-10, TNF-α, and PGE_2_ was measured using ELISA kits, according to the manufacturer’s instructions.

### 2.7. Phagocytosis Assay

The phagocytic activity was determined using a phagocytosis assay kit, in accordance with the manufacturer’s instructions. Briefly, the cells were seeded on 4-well chamber slide (SPL Life Sciences Co., Pocheon, Republic of Korea) at a density of 2 × 10^5^ cells/well, and incubated for 24 h. For subsequences, the cells were treated with 0.5 or 1.0 mg/mL GSH, or LPS (1 ng/mL), and then fluorescently labeled Latex Beads-Rabbit IgG-FITC complex was added to each well. After incubation for 2 h, the cells were washed with assay buffer, and treated with 40 μM 4′,6-Diamidino-2-phenylindole (DAPI) for 10 min at 37 °C in 5% CO2 atmosphere. The fluorescence intensity was measured by fluorescence microscopy (Leica Microsystems, Wentzler, Germany) and flow cytometer (BD Biosciences, San Jose, CA, USA).

### 2.8. Measurement of Intracellular ROS Generation

The production of intracellular ROS was measured using ROS-sensitive fluorescent dye, DCF-DA, as previously described [26]. To determine the amount of ROS generation by flow cytometer and fluorescence microscopy, the cells were seeded into 6-well plate and 4-well slide chamber, respectively. After incubation of 24 h, the cells were pretreated with the indicated concentrations of GSH for 1 h, and then stimulated with LPS (1 ng/mL) for 6 h. In the last 20 min of treatment, 10 μM DCF-DA was added to the incubated cells in dark environment. For the assessment of ROS production by flow cytometer, the cells were washed twice with calcium- and magnesium-free Dulbecco’s phosphate-buffered saline (DPBS), and 10,000 events were immediately analyzed using a flow cytometer (BD Biosciences) at 480 nm/520 nm. To observe the ROS generation by fluorescence microscopy, the cells were washed twice with DPBS, and then fixed with 4% paraformaldehyde (pH 7.4) for 20 min. The fixed cells were washed twice with PBS, and analyzed by fluorescence microscopy (Carl Zeiss).

### 2.9. Western Blot Analysis

As described previously, total protein was extracted from the cells using the Bradford Protein assay kit [27]. The nuclear protein extracts obtained from the cells were prepared using the NE-PER Nuclear and Cytoplasmic Extraction Reagents (Thermo Fisher Scientific Inc., Waltham, MA, USA). An equal amount of protein from the samples was separated by 10–13% sodium-dodecyl sulfate gel electropThermoTherhoresis, and transferred onto polyvinylidene difluoride membranes (Schleicher & Schuell, Keene, NH, USA). The membranes were blocked with 5% non-fat skim milk in Trisbuffered saline containing 0.1% Triton X-100 (TBST) for 1 h, and probed with specific primary antibodies, which were purchased from Santa Cruz Biotechnology, Inc. (Santa Cruz, CA, USA), Merck Millipore (Temecula, CA, USA), BD Biosciences (Franklin Lakes, NJ, USA), and Abcam Inc. (Cambridge, UK), at 4 °C overnight. After washing 3 times with TBST, the membranes were incubated with the appropriate HRP-conjugated secondary antibodies (Santa Cruz Biothechnology, Inc.,) for 2 h at RT. The expression of protein was detected by enhanced chemiluminescence kit (GE Healthcare Life Sciences, Little Chalfont, UK), and visualized by Fusion FX Image system (Vilber Lourmat).

### 2.10. Statistical Analysis

All experiments were performed at least three times. Data were analyzed using GraphPad Prism software (version 5.03; GraphPad Software, Inc., La Jolla, CA, USA), and expressed as the mean ± standard deviation (SD). Differences between groups were assessed using analysis of variance followed by ANOVA-Tukey’s post hoc test, and *p* < 0.05 was considered to indicate a statistically significant difference.

## 3. Results

### 3.1. Effects of GSH on the Cell Viability of RAW 264.7 Macrophages

**Figure 1:**
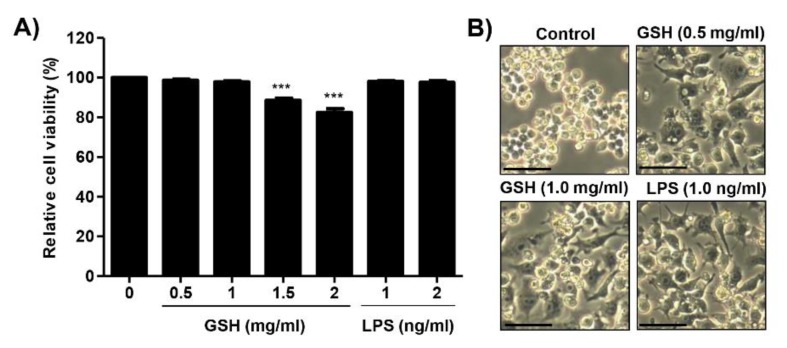
Effects of reduced glutathione on the cell viability in RAW 264.7 macrophages. Cells were treated with different concentration of GSH (0.5 to 2 mg/mL) and LPS of 1 and 2 ng/mL for 24 h. (**A**) Cell viability was measured by MTT assay. Data are expressed as the mean ± SD (*n* = 3). The statistical analyses were conducted using analysis of variance (ANOVA-Tukey’s post hoc test) between groups. *** *p* < 0.001 when compared to control. (**B**) The representative morphological changes of cells were taken using an inverted microscope (Scale bar; 20 μm).

Cell viability of RAW 264.7 cells after incubation with GSH for 24 h was estimated with MTT assay. GSH did not affect the viability in RAW 264.7 cells up to 1 mg/mL, but showed slight cytotoxicity at concentration of over 1.5 mg/mL (Figure 1A). LPS as positive control had no cytotoxicity up to 2.0 ng/mL. Figure 1B shows that the morphology of the control cell appeared a round form, whereas GSH and LPS changed to an activated irregular form with spindle-shaped cells. Based on these results, the immunomodulatory activity of GSH at concentration of up to 1 mg/mL in RAW 264.7 cells was assessed.

### 3.2. GSH Increased MP-Derived Cytokines and Chemokines

**Figure 2:**
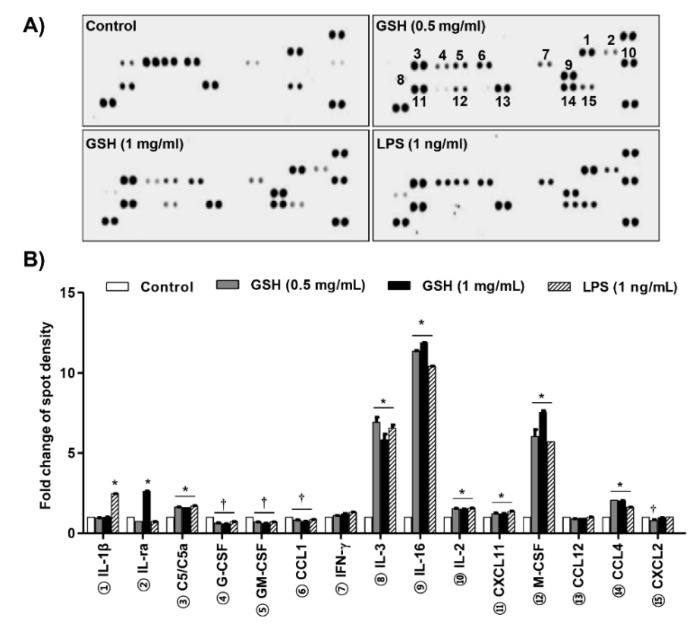
Effects of GSH on MP-derived cytokines and chemokines. Protein array analysis demonstrating the effects of GSH on cytokine profile. Cells were treated with the indicated concentrations of GSH (0.5 to 2 mg/mL) and LPS of 1 and 2 ng/mL for 24 h. The supernatants were then analyzed using the cytokine array. (**A**) Spots with the most prominent differentially regulated cytokines are identified by circles. (**B**) Quantitative analysis of spots on the cytokine array membrane. Quantitative analysis of mean pixel density was performed using the ImageJ^®^ software, and data are the mean ± SD of three independent experiments. * *p* < 0.05 and † *p* < 0.05 indicate up-regulation and down-regulation of significant differences compared to control group, respectively.

**Figure 3:**
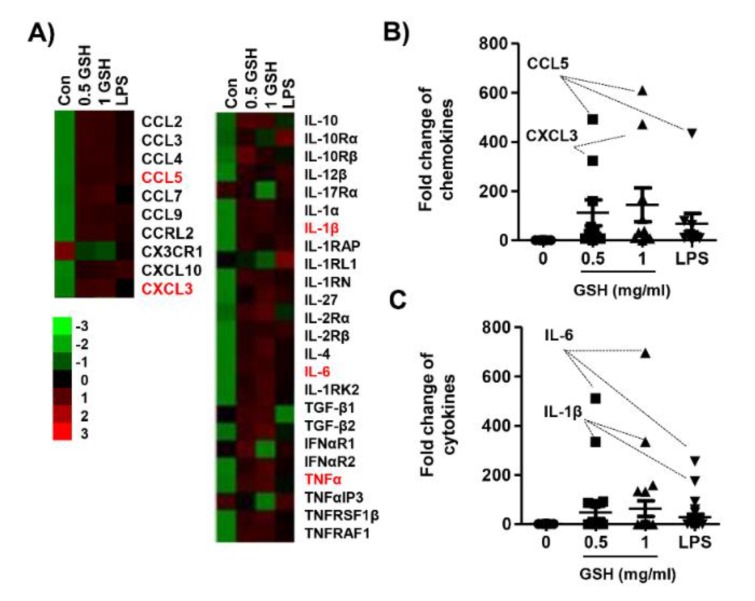
Heatmap of candidate gene expression for MP using NanoString nCounter^®^ miRNA Expression Assays. The cells were treated with GSH or LPS, and then incubated for 24 h. Total RNA was collected by collecting the cells, and hybridization was performed using a reporter probe and a capture probe. After digital analysis through nCounter nanostring assay (NCT-120), the raw data was normalized using the housekeeping gene, and the gene expression change was represented by the fold change value. (**A**) Heatmap representing differentially expressed genes with fold-change cutoff of 0.5 and 2 (red and green, respectively). (**B**) and (**C**) Expression of each gene was indicated as fold change compared with control. Table 1 shows the abbreviations and designations defined.

**Figure 4:**
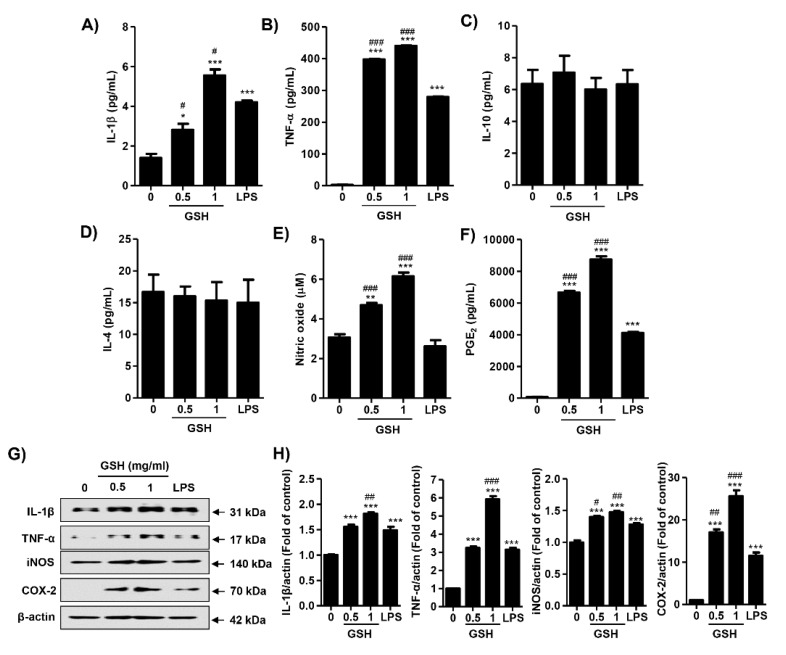
Effects of GSH on most-potent cytokines, NO and PGE_2_ production in RAW 264.7 macrophages. Cells were treated with GSH of 0.5 to 1 mg/mL and 1 ng/mL LPS, and then incubated for 24 h. The production of IL-1β (**A**), TNF-α (**B**), IL-4 (**C**) and IL-10 (**D**) on cell supernatant were measured by ELIAS kits. (**E**) The amounts of NO were measured using the Griess reagent in culture supernatant. (**F**) The levels of PGE_2_ were measured by an ELIAS kit. (**G**) The cell lysates were immunostained for IL-1β, TNF-α, iNOS, and COX-2. Actin was used as an internal control. Images of the membranes were photographed with the Fusion Fx image acquisition system. (**H**) Relative band density was measured by ImageJ. All data are expressed as the mean ± SD (*n* = 3). The statistical analyses were conducted using analysis of variance (ANOVA-Tukey’s post hoc test) between groups. * *p* < 0.05 ** *p* < 0.01 and *** *p* < 0.001 indicates significant difference compared to the non-treated control group. # *p* < 0.05, ## *p* < 0.01 and ### *p* < 0.001 when compared to LPS treatment.

To assess the effect of GSH on MP, we determined the expression of different 40 cytokines after 24 h in response to treatment with GSH. Figure 2A,B show that GSH significantly increased the expression of cytokines, namely IL-3, IL-16, IL-2, CXCL11, M-CSF, and CCL4 (*p* < 0.05 compared with control). In contrast, GSH slightly decreased the expression of G-CSF, GM-CSF, and CCL-1, and CXCL2. In addition, we monitored the expression of MP-related genes using nCounter Sprint platform (Figure 3A). According to the analysis for up to 800 candidate genes, we found that GSH markedly up-regulated chemokine ligands, including CCL5 and CCL13 (Figure 3B). Furthermore, we also identified that GSH induced the over-expression of IL-1β, IL-6, and TNF-α in RAW 264.7 macrophages (Figure 3C). Table 1 indicates the name and description of the genes with significant difference compared to control. We reconfirmed the expression of the most potent cytokines, including IL-1β, TNF-α, IL-4 and IL-10 using ELISA kit and immunoblotting. Figure 4A shows that GSH significantly induced the production of IL-1β compared with control in a dose-dependent manner (0.5 mg/mL GSH: 2.81 ± 0.54 pg/mL; 1 mg/mL GSH: 5.55 ± 0.52 pg/mL). In addition, 0.5 and 1 mg/mL GSH up-regulated the production of TNF-α to 397.49 ± 5.84 and 439.93 ± 3.94 pg/mL, respectively (Figure 4B). Meanwhile, LPS as a positive control also significantly up-regulated the production of IL-1β (4.20 ± 0.14 pg/mL) and TNF-α (279.24 ± 4.77 pg/mL) compared with control, although the levels were lower than for 1 mg/mL GSH. As shown in the result of immunoblot analysis, the effect of GSH on the expression of the most potent cytokines was similar to ELISA. GSH induced the overexpression of IL-1β (1 mg/mL GSH: 1.76 fold of control) and TNF-α (1 mg/mL GSH: 1.60 fold of control) compared with the control, and LPS also slightly increased the expression of these cytokines (Figure 4G,H). However, we observed the levels of putative M2 marker including IL-4 and IL-10 were no changed with GSH or LPS (Figure 4C,D) in RAW264.7 cells. Additionally, we confirmed the effect of GSH on the MP-derived cytokines in human monocytic cell lines including THP-1 and U-937. In common with results from RAW 264.7 cells, the levels of cellular TNFα and expression of inflammatory mediators were up-regulated by GSH treatment in THP-1 and U-937 cells, but the levels of M2 markers (i.e., IL-4 and IL-10) were not changed by GSH (supplementary Figure S1C–F).

### 3.3. GSH Induces the Production of NO and PGE_2_

Cells were treated with GSH of 0.5 to 1 mg/mL and 1 ng/mL LPS, and then incubated for 24 h. We measured whether GSH modulated the production of NO and PGE_2_. Figure 4E shows that GSH treatment significantly enhanced the amount of NO in a dose-dependent manner (0.5 mg/mL GSH: 4.69 ± 0.20 μM, 1.53-fold of control; 1 mg/mL GSH: 6.15 ± 0.32 μM, 2.01-fold of control), but no change was observed in LPS treatment. In addition, above the GSH concentration of 0.5 mg/mL, the secretion of PGE_2_ was also markedly up-regulated to 6656.52 ± 226.83 pg/mL in a dose-dependent manner (Figure 4F). LPS also significantly increased the levels of PGE_2_ (4118.76 ± 114.26 pg/mL), but the levels were lower than the efficacy of 0.5 mg/mL GSH. Then, we examined the expression of NO inducible enzyme, inducible NO synthase (iNOS), and PGE_2_ synthesis enzyme, cyclooxygenase-2 (COX-2). GSH slightly increased the protein expression of iNOS (1 mg/mL GSH: 1.47 fold of control), and substantially overexpressed COX-2 (1 mg/mL GSH: 25.61 fold of control, Figure 4E,G,H). The results of immunoblot analysis have something in common with Figure 4C,D. Meanwhile, GSH also increased the levels of NO in THP-1 and U-937 cells, human monocytic cell lines, in the same manner with RAW 264.7 cells (supplementary Figure S1B).

### 3.4. GSH Activated the Phagocytosis of Macrophages

**Figure 5:**
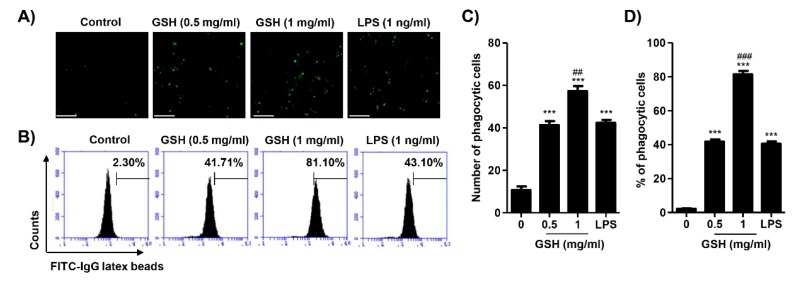
Effects of GSH on phagocytosis activity in RAW 264.7 macrophages. Cells were treated with GSH of 0.5 to 1 mg/mL and 1 ng/mL LPS, and for 24 h. (**A**) The phagocytosis activity was visualized by fluorescence microscopy. Scale bar; 200 μm. (**B**) The fluorescence intensity was counted and indicated as the number of phagocytic cells per field of view. (**C**) The phagocytosis capacity of GSH was gauged by flow cytometer. The images shown are representative of at least three independent experiments. (**D**) The statistical analyses were conducted using analysis of variance (ANOVA-Tukey’s post hoc test) between groups. *** *p* < 0.001 when compared to control. ## *p* < 0.01 and ### *p* < 0.001 when compared to LPS treatment.

To assess the effect of GSH on phagocytosis, we performed a fluorescence-activated cell sorting (FACS)-based assay and a fluorescence microscopy observation. Figure 5A,C show that GSH significantly increased the numbers of phagocytic cells to 3.88-fold (0.5 mg/mL GSH) and 5.38-fold (1 mg/mL GSH) of control in RAW 264.7 macrophages, respectively. In addition, as the result of FACS-based assay, we reconfirmed that GSH markedly enhanced the phagocytic cells (0.5 mg/mL GSH: 41.77 ± 2.20%; 1 mg/mL GSH: 81.37 ± 1.57%) (Figure 5B,D). LPS also up-regulated the phagocytosis activity, and the levels were similar to those of the 0.5 mg/mL GSH treatment.

### 3.5. GSH Suppressed LPS-Induced Intracellular ROS Generation

**Figure 6:**
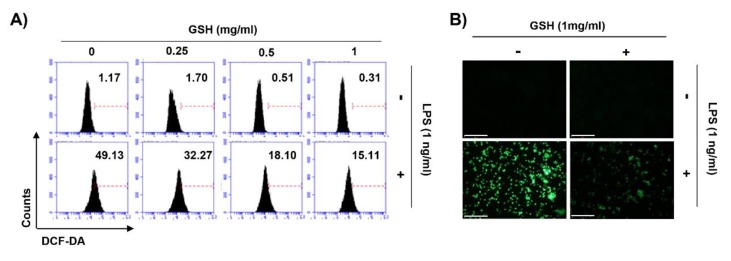
Effects of GSH on intracellular ROS generation in RAW 264.7 macrophages. Cells were pretreated with various concentrations of GSH for 1 h, and then stimulated with LPS (1 ng/mL) for 6 h. (**A**) After staining with DCF-DA, DCF fluorescence was monitored by flow cytometer. Results are presented as the means of two independent experiments. (**B**) Images were obtained by fluorescence microscopy (scale bar; 200 μm). The images shown are representative of at least three independent experiments.

We investigated the effect of GSH on ROS generation estimated during the phagocytosis of macrophage. To evaluate the intracellular ROS generation, we performed FACS-based assay using DCF-DA fluorescence dye. Figure 6A shows that GSH treatment did not change the intracellular ROS generation, following by concentration rise (0.25 to 1 mg/mL). In contrast, LPS significantly increased ROS generation to 49.13%. Based on these results, we assessed whether the potent anti-oxidative effect of GSH contributes to the scavenging of ROS in LPS-stimulated macrophages. As a result, GSH markedly suppressed the production of ROS in LPS-stimulated RAW 264.7 cells, in a dose-dependent manner (Figure 6A). Additionally, we reconfirmed that LPS strongly enhanced the production of intracellular ROS under fluorescence microscopy (Figure 6B). However, GSH markedly suppressed the increase of ROS generation, followed by LPS.

### 3.6. GSH Activated Nrf2 and NF-κB Signaling Pathways

**Figure 7:**
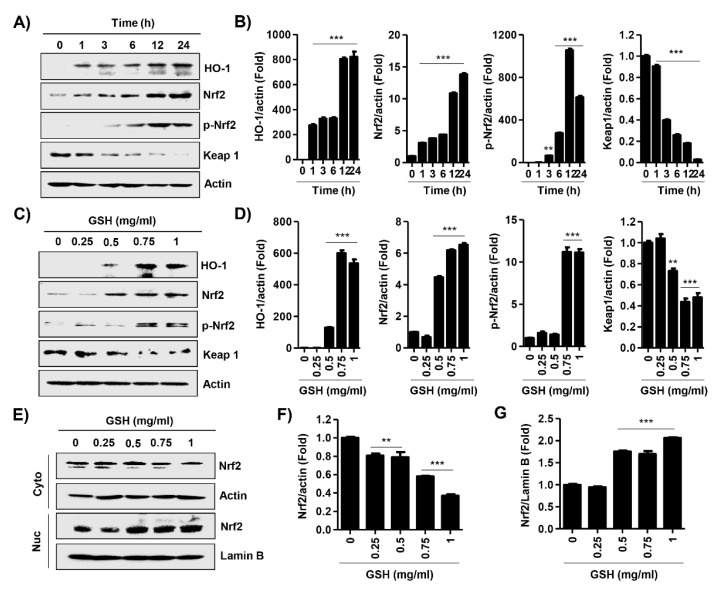
Effects of GSH on Keap1/Nrf2 activation in RAW 264.7 macrophages. (**A**,**C**) Cells were incubated with 1 mg/mL GSH for the indicated periods, or with the indicated concentration of GSH for 24 h. Expression of Nrf2, HO-1, and Keap1 was determined by Western blot analysis with total cell lysates. Actin was used as an internal control. (**B**,**D**) Relative band density was measured by ImageJ. (**E**) Cells were incubated with the indicated concentration of GSH for 24 h. Expression of Nrf2 was determined by Western blot analysis with cytosol and nuclear fraction. Actin and Lamin B were used as an internal control for cytosol and nuclear, respectively. (**F**,**G**) Relative band density for Nrf2 expression of cytosol and nuclear fraction was measured by ImageJ. All data are the means ± SD (*n* = 3). The statistical analyses were conducted using analysis of variance (ANOVA-Tukey’s post hoc test) between groups. ** *p* < 0.01 and *** *p* < 0.001 indicates significant difference compared to the non-treated control group.

**Figure 8:**
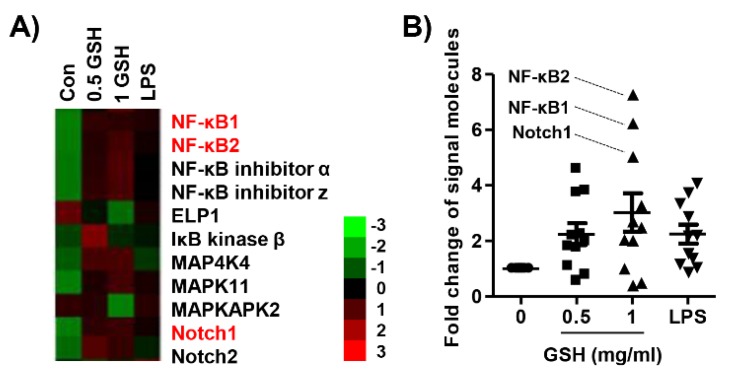
Effects of GSH on NF-κB signaling systems in RAW 264.7 macrophages. Cells were treated with GSH of 0.5 to 1 mg/mL and 1 ng/mL LPS, and for 24 h. Total RNA was collected by collecting the cells, and hybridization was performed using a reporter probe and a capture probe. After digital analysis through nCounter nanostring assay (NCT-120), the raw data was normalized using the housekeeping gene, and the gene expression change was represented by the fold change value. (**A**) Heatmap representing differentially expressed genes with fold-change cutoff of 0.5 and 2 (red and green, respectively). (**B**) The expression of each gene was indicated as fold change compared with control. Table 1 shows the abbreviations and designations defined.

We performed an immunoblot analysis for the effect of GSH on the Nrf2 signal pathway. Figure 7A,C show that the expression and phosphorylation of Nrf2 were also up-regulated in the GSH-treated cell. In contrast, GSH gradually decreased the expression of Kelch-like ECH associated protein 1 (Keap1), a repressor protein that binds to Nrf2, and promotes its degradation by the ubiquitin proteasome pathway [28,29]. Moreover, GSH induced the gradually increase of antioxidant enzyme heme oxygenease-1 (HO-1), one of the Nrf2-dependent cytoprotective enzymes, in a time- and dose-dependent manner. Furthermore, GSH showed statistically significantly induced nuclear accumulation of Nrf2 (Figure 7E). In addition, we monitored the expression of immune-related signal genes using the nCounter analysis system. Table 1 indicates the name and description of those genes with significant difference compared with control. Consequently, we confirmed that GSH up-regulated the expression of mitogen-activated protein kinase (MAPK) and Notch signal genes, as well as NF-κB signal genes in RAW 264.7 macrophages (Figure 8).

## 4. Discussion

Macrophages function as a critical regulator in multiple biological events, including reproduction, vasodilation, angiogenesis, malignancy, and immune response [30]. In response to various stimulators or pathophysiologic conditions, macrophages can adopt different function as M1 and M2 macrophages, and that induced phenotype switch between macrophage subsets [8,31,32]. The phenotype transition is involved alteration of the macrophage transcriptome and regulatory networks [33]. In the response to pathogens, M1 macrophages come to the wounded place, and acts as a host defense, and elicits an immune system through phagocytosis, antigen presentation, and cytokine secretion [3]. Then M2 macrophages are recruited to repair tissue and heal the wound [34]. To avoid the recruitment of monocyte/macrophages, M1 can switch the phenotype to M2 [35]. Under healthy condition, macrophages remain in an M1/M2 balanced state. In contrast, dysregulation of MP, which results in disease progression, and may trigger tumor progression under physiological condition [36,37]. Therefore, the stimulation of the immune system via regulation to M1 polarized macrophages is one of the significant approaches to increase the human defense system. Based on this theory, many scientists are concerned for new materials that have immune-enhancing activity through M1-mediated pro-inflammatory cytokines and chemokines, and this has become an important research theme in the field of cancer prevention and immunopharmacology [38,39,40,41]. In the present study, we estimated the immune-modulatory effect of GSH, known as a potent antioxidant. We also assessed the effect of GSH on M1-derived pro-inflammatory mediator in RAW 264.7 cells. Our results show that GSH induced the macrophage activation up to 1 mg/mL treatment (Figure 1), as well as up-regulated pro-inflammatory cytokines, including IL-1β, IL-6, IL-3, IL-16, and TNF-α, as results of the cytokine array and nCounter gene array (Figure 2 and Figure 3). Furthermore, we confirmed GSH significantly stimulated the secretion and expression of the most potent M1-associated cytokine IL-1β and TNF-α by ELISA and immunoblot analysis (Figure 4A,B,G). These cytokines are well known to play a central role in mediating the inflammation and immune regulatory signal (Table 1). However, GSH no effect on the levels of Th2 cytokines IL-4 and IL-10 that induced M2 polarization. Many studies suggested that immune-enhancing compounds induce the secretion of these pro-inflammatory cytokines [38,39,40,41]. Especially, endogenous pyrogens including IL-1β are released in response to pathogen at the early stage of the immune reaction. [31]. It also has the capability to promote phagocytosis, which stimulates macrophage proliferation, and induces leukocyte migration [42]. Similar to IL-1 β, TNF-α is also an important pro-inflammatory cytokine, which is one of the first to be released in response to a pathogen, and stimulated the acute phase of the immune response by activating macrophages [43]. In concert with IL-17, TNF-α induces the secretion of neutrophil-attracting chemokines and cell adhesion molecules [5,6,7,8,44,45]. In the present study, we found that GSH overexpressed neutrophil-attracting chemokines, such as CCL4, and M-CSF, as a result of the cytokine array (Figure 2A,B). Furthermore, we confirmed that CCL5 and CXCL13 showed conspicuous alteration by GSH as a result of nCounter gene expression assay (Figure 3A,B). These chemokines are inflammatory chemoattractants for T cells, basophils, eosinophils, and dendritic cells [31]. Moreover, they are well known to play a central role in mediating the inflammation and immune regulatory signal Table 1 Consequently, our results have demonstrated that GSH enhanced the secretion and expression of pro-inflammatory cytokines and chemokines, which involve M1-mediated immune response, whereas no effect on M2-related cytokines.

As one of the immune cells, macrophages promote the inflammatory responses by not only producing pro-inflammatory cytokines, but also mediators, such as NO and PGE_2_ [46,47]. NO is a toxic defense molecule that acts as an effector of the host innate immune response [48]. In addition, macrophages inhibit pathogen replication by releasing numerous cellular molecules, including NO [48]. NO also contributes to the physiologic and pathophysiologic process, including the growth and death of many immune and inflammatory cell types. Zhao et al. [49] reported that intracellular GSH prevented cell apoptosis via NO in vascular smooth muscle cells. Trimenstein et al. [50] also identified the production of NO dependent on PGE_2_ levels in hepatocytes. Another pro-inflammatory mediator, PGE_2_ has an important role in the regulation of cellular immune response, such as T cell proliferation, lymphokine production, and cytotoxicity [51]. It is generated from arachidonate by the action of COX-2, which is a more critical source of prostanoid formation in inflammation [52]. Figure 4F shows that GSH markedly enhanced the secretion of PGE_2_ in a dose-dependent manner, and at levels higher than LPS as positive control. The increase of PGE_2_ levels resulted from the up-regulation of COX-2 (Figure 4G). Similar to these results, our findings show that GSH significantly enhanced the secretion of NO in a dose-dependent manner through the up-regulation of iNOS in RAW 264.7 macrophages (Figure 4E,G). This result proves the GSH can have immune-enhancing activity by increasing NO and PGE_2_, as effectors of the host defense system.

The transcription factor NF-κB promotes immune response by the regulation of inflammatory gene expression [53]. In response to cytokines produced by lymphocytes and other immune cells, NF-κB-dependent differentiation of MP is an important factor in the progress of inflammation [53]. Numerous studies have demonstrated that the activation of NF-κB leads to the signaling pathways, such as MAPKs, that play a critical role in the regulation of inflammatory responses [54,55]. Sen et al. also reported that TNF-α induced the activation of NF-κB associated with cytoplasmic GSH levels [56]. Therefore, we performed screening of NF-κB-related signaling genes through the nCounter Sprint platform. Our results showed that GSH up-regulated not only the expression of NF-κB subunits, including NF-κB1 and NF-κB2, but also MAPK family members (Figure 8). Interestingly, GSH also increased the expression of Notch 1 and 2 in the present study. Several studies have reported that Notch-1 is interrelated with NF-κB, and both pathways synergistically modulate the pro-inflammatory function [57,58,59]. Although further studies should be conducted to confirm the up- and down-streams of NF-κB signaling, these findings suggest that the immune-modulatory efficacy of GSH is associated with the NF-κB, MAPK, and Notch signaling pathways.

Phagocytosis is crucial to a variety of cellular biological functions, including tissue remodeling, and the continuous clearance of dying cells. Moreover, activated macrophages are characterized by phagocytosis, which represent essential response to the host defense against pathogens [60]. Therefore, we evaluated whether GHS regulates phagocytosis in RAW 264.7 macrophages. Our results showed that 0.5 mg/mL GSH significantly stimulated the number of phagocytic cells and phagocytosis, and at levels similar to that of the positive control. Additionally, the capacity of phagocytosis is markedly increased by GSH in a dose-dependent manner (Figure 5). There are well-known that phagocytes produce ROS during phagocytosis, and subsequently eliminate pathogens by ROS-mediated microbial DNA damage [61,62]. Namely, ROS have a biocidal effect on infiltrated pathogens, and they can also injure the cells of the host [63]. In the present study, GSH did not change intracellular ROS generation during phagocytosis, but LPS markedly increased it (Figure 6A). However, GSH treatment significantly decreased LPS-induced ROS generation in a dose-dependent manner (Figure 6A,B). As is well known, the increase of ROS by LPS was due to the activation of Toll-like receptor 4 (TLR4) cascade, but was suppressed by GSH. This finding suggests that GSH is the most abundant antioxidant and major detoxification agent for cell defense against ROS, unlike immunological ROS generation [64]. In other words, our results indicate that GSH plays a role in the cell defense against ROS. Based on this result, we investigated the alteration of transcription factors related to the ROS scavenging capacity of GSH. HO-1 has antioxidant and cytoprotective properties, and play a key role in the innate immunity [65,66]. Previous report described that HO-1 modulated innate immunity by attenuated the TLR4-mediated signaling [67]. In addition, there are well-known that the activation of Nrf2 enhances HO-1 expression in several cell types [66]. Nrf2 binds to Keap1 in its resting state, but it is dissociated in Keap1 when exposed to electrophiles or other mediators [68]. In present study, we found that GSH increased nuclear accumulation of Nrf2, while decreasing Keap1 expression, and this process was accompanied by increased expression of HO-1 (Figure 7). The notable here is that Nrf2 plays a critical regulator in modulating the intracellular GSH level. Several scientists have confirmed that Nrf2 is important in maintaining intracellular GSH levels and HO-1 in redox homeostasis [69,70,71]. Our previous report [72] and Song et al.’s report [73] also demonstrated that GSH activates Nrf2-mediated signal pathways to protect cells from oxidative stress. Furthermore, increasing evidences described that GSH-mediated redox-status regulates Th1/Th2 balance in innate immune response [16,74]. Peterson et al. reported that GSH depletion attenuated Th1-mediated cytokine production and induced Th2-mediated response [71]. In current study, our findings shown that GSH mediates the induction of HO-1 through the regulation of Keap1-Nrf2 pathway, which may suggests contribute to eliminating ROS. However, further studies are required to identify the GSH depletion-mediated molecular mechanisms on MP and the role of endogenous GSH involved on MP.

## 5. Conclusions

Overall, we have shown that GSH induced macrophage activation and M1 polarization in the RAW 264.7 cell model. In brief, GSH stimulated M1-associated immune response, including pro-inflammatory cytokines, chemokines, inflammatory mediators, and phagocytosis. The immune-modulatory effect of GSH was associated with NF-κB, MAPKs, and Notch signal pathways. Furthermore, GSH has potential ROS scavenging ability through activation of the Keap1-Nrf2 signaling-mediated HO-1. In addition, GSH may exhibit immune-enhancing activity by the modulation of M1-like MP, and such efficacy of GSH may be a useful strategy to increase the human defense system.

## Figures and Tables

**Table 1:** Name and description of the genes.

Gene	Symbol	Function (NCBI Gene Database)
Interleukin 1 alpha	IL-1α	This cytokine is a pleiotropic cytokine involved in various immune responses, inflammatory processes, and hematopoiesis. This cytokine is produced by monocytes and macrophages as a proprotein, which is proteolytically processed, and released in response to cell injury, and thus induces apoptosis.
Interleukin 1 beta	IL-1β	This cytokine is an important mediator of the inflammatory response, and is involved in a variety of cellular activities, including cell proliferation, differentiation, and apoptosis.
Interleukin 3	IL-3	This cytokine is capable of supporting the proliferation of a broad range of hematopoietic cell types. It is involved in a variety of cell activities, such as cell growth, differentiation, and apoptosis.
Interleukin 4	IL-4	This cytokine is a ligand for interleukin 4 receptor. The interleukin 4 receptor also binds to IL13, which may contribute to many overlapping functions of this cytokine and IL13. STAT6, a signal transducer and activator of transcription, has been shown to play a central role in mediating the immune regulatory signal of this cytokine.
Interleukin 6	IL-6	This gene encodes a cytokine that functions in inflammation and the maturation of B cells. In addition, the encoded protein has been shown to be an endogenous pyrogen capable of inducing fever in people with autoimmune diseases or infections.
Interleukin 12	IL-12	This gene encodes a subunit of interleukin 12, a cytokine that acts on T and natural killer cells, and has a broad array of biological activities. This cytokine is expressed by activated macrophages that serve as an essential inducer of Th1 cells development.
Interleukin 16	IL-16	The cytokine function is exclusively attributed to the secreted C-terminal peptide, while the N-terminal product may play a role in cell cycle control. Caspase 3 is reported to be involved in the proteolytic processing of this protein.
Tumor necrosis factor-alpha	TNF-α	This gene encodes a multifunctional proinflammatory cytokine. This cytokine is involved in the regulation of a wide spectrum of biological processes, including cell proliferation, differentiation, apoptosis, lipid metabolism, and coagulation.
Chemokine (C-X-C motif) ligand 1	CXCL1	This protein plays a role in inflammation, and as a chemoattractant for neutrophils.
Chemokine (C-X-C motif) ligand 2	CXCL2	This antimicrobial gene is part of a chemokine superfamily that encodes secreted proteins involved in immunoregulatory and inflammatory processes.
Chemokine (C-X-C motif) ligand 3	CXCL3	This protein plays a role in inflammation, and as a chemoattractant for neutrophils.
TNF alpha induced protein 3	TNFAIP3	This gene was identified as a gene whose expression is rapidly induced by the tumor necrosis factor (TNF). The protein has been shown to inhibit NF-kappa B activation, as well as TNF-mediated apoptosis.
TNF receptor superfamily member 11 alpha	TNFRSF11A	This receptor can interact with various TRAF family proteins, through which this receptor induces the activation of NF-kappa B and MAPK8/JNK.
Chemokine (C-X-C motif) ligand 11	CXCL11	Chemokines also play fundamental roles in the development, homeostasis, and function of the immune system, and they have effects on cells of the central nervous system, as well as on endothelial cells involved in angiogenesis or angiostasis.
Macrophage colony stimulating factor	MCS-F	The protein encoded by this gene is a cytokine that controls the production, differentiation, and function of macrophages.
Chemokine (C-C motif) ligand 2	CCL2	Chemokines are a superfamily of secreted proteins involved in immunoregulatory and inflammatory processes.
Chemokine (C-C motif) ligand 3	CCL3	This locus represents a small inducible cytokine. The encoded protein, also known as macrophage inflammatory protein 1 alpha, plays a role in inflammatory responses through binding to the receptors CCR1, CCR4, and CCR5
Chemokine (C-C motif) ligand 4	CCL4	The encoded protein is secreted, and has chemokinetic and inflammatory functions.
Chemokine (C-C motif) ligand 7	CCL7	This gene encodes monocyte chemotactic protein 3, a secreted chemokine, which attracts macrophages during inflammation and metastasis.
Chemokine (C-C motif) receptor-like 2	CCRL2	Chemokines and their receptors mediated signal transduction are critical for the recruitment of effector immune cells to the site of inflammation. This gene is expressed at high levels in primary neutrophils and primary monocytes, and is further upregulated on neutrophil activation, and during monocyte to macrophage differentiation.
Nuclear factor kappa B subunit 1	NFκB1	This gene encodes a 105 kD protein which can undergo cotranslational processing by the 26S proteasome to produce a 50 kD protein. This protein is a Rel protein-specific transcription inhibitor, and the 50 kD protein is a DNA binding subunit of the NF-B (NFKB) protein complex.
Nuclear factor kappa B subunit 2	NFκB2	This gene encodes a subunit of the transcription factor complex NF-B. The NF-B complex is expressed in numerous cell types, and functions as a central activator of genes involved in inflammation and immune function.
Mitogen-activated protein kinase kinase kinase kinase 4	MAP4K4	This kinase has been shown to specifically activate MAPK8/JNK, and mediate the TNF-alpha signaling pathway.
Mitogen-activated protein kinase 11	MAPK11	This gene encodes a member of a family of protein kinases that are involved in the integration of biochemical signals for a wide variety of cellular processes, including cell proliferation, differentiation, transcriptional regulation, and development.
Notch homolog 1	Notch1	Notch signaling is an evolutionarily conserved intercellular signaling pathway. This receptor plays a role in the development of numerous cell and tissue types. Mutations in this gene are associated with aortic valve disease, Adams-Oliver syndrome, T-cell acute lymphoblastic leukemia, chronic lymphocytic leukemia, and head and neck squamous cell carcinoma.
Notch homolog 2	Notch2	Notch family members play a role in a variety of developmental processes by controlling cell fate decisions. The Notch signaling network is an evolutionarily conserved intercellular signaling pathway, which regulates interactions between physically adjacent cells.

Name and description of the genes with a significant difference compared with control (*p* < 0.05).

## Supplementary Materials

The following are available online at https://www.mdpi.com/2076-3921/8/9/413/s1, Figure S1: Effect of GSH on M1-associated immune response in THP-1 and U-937 cells.
