# Supplementary material for: Glutathione Induced Immune-Stimulatory Activity by Promoting M1-Like Macrophages Polarization via Potential ROS Scavenging Capacity
Source: Antioxidants (Basel). 2019 Sep 18;8(9):413. doi: 10.3390/antiox8090413 (PMC6770173; doi:10.3390/antiox8090413)
Supplement: Supplementary file 1 [file antioxidants-08-00413-s001.zip › Supple Figure legend.docx]

**Supplementary Figure S1. Effect of GSH on M1-associated immune response in THP-1 and U-937 cells.** Cells were treated with GSH of 0.5 to 1 mg/ml and 1 ng/ml LPS, and then incubated for 24 h. (A) Cell viability was measured by MTT assay. The production of nitric oxide (B), TNF-α (C), IL-4 (D) and IL-10 (E) on cell supernatant were measured by ELISA kit. All data are expressed as the mean ± SD (n=3). The statistical analyses were conducted using analysis of variance (ANOVA-Tukey’s post hoc test) between groups. *p<0.05 **p<0.01 and ***p<0.001 indicates significant difference compared to the non-treated control group. #p<0.05 and ##p<0.01 when compared to LPS treatment. (F) The cell lysates were immunostained for indicated antibodies. Images of the membranes were photographed with the Fusion Fx image acquisition system.
