# Supplementary figures and images for: Glutathione Induced Immune-Stimulatory Activity by Promoting M1-Like Macrophages Polarization via Potential ROS Scavenging Capacity
Source: Antioxidants (Basel). 2019 Sep 18;8(9):413. doi: 10.3390/antiox8090413 (PMC6770173; doi:10.3390/antiox8090413)

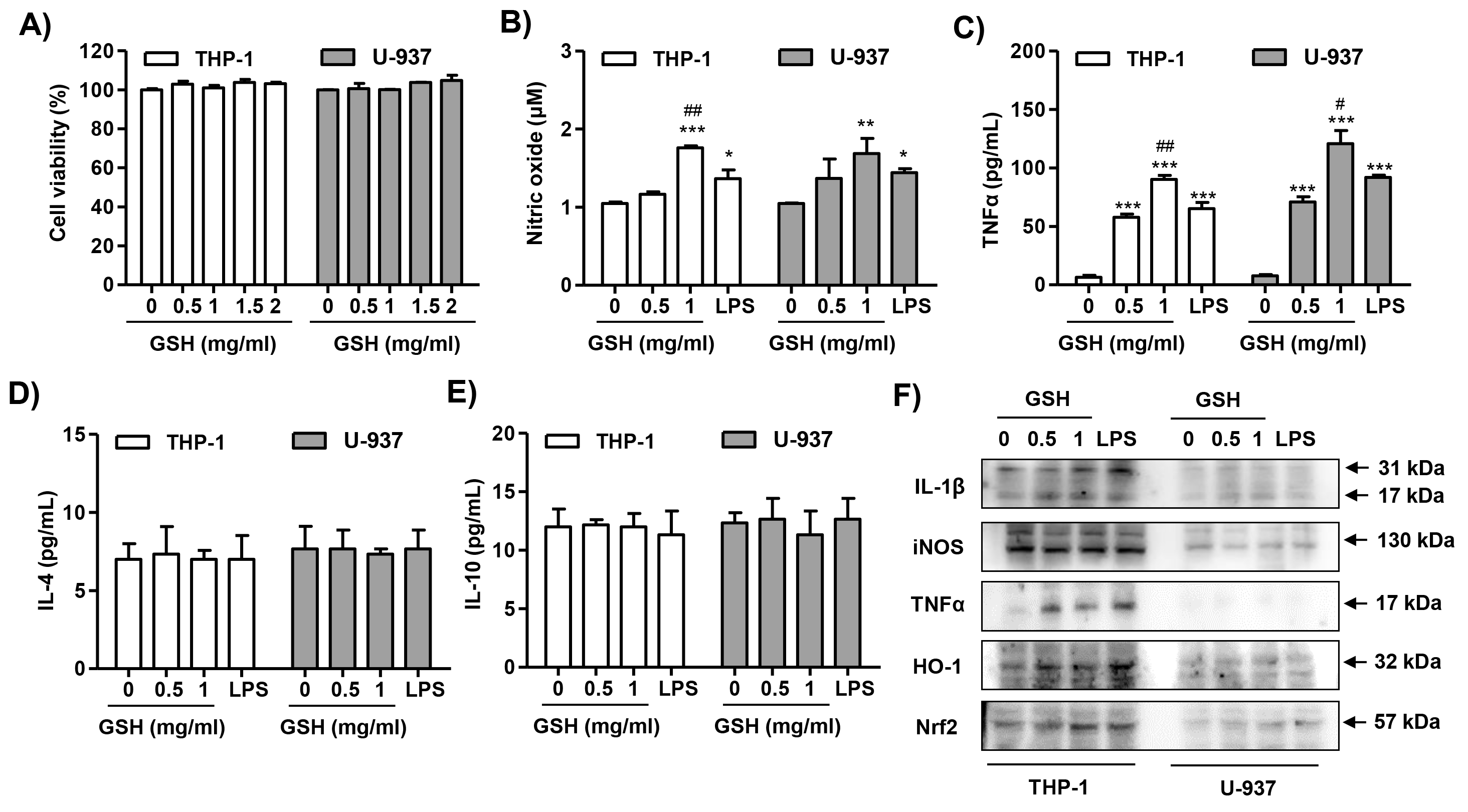

Supplement: Supplementary file 1 [file antioxidants-08-00413-s001.zip › Figures S1-R1.tif]
